# Side lying during nebulisation can significantly improve apical deposition in healthy adults and adults with mild cystic fibrosis lung disease: a randomised crossover trial

**DOI:** 10.1186/s12890-019-0886-7

**Published:** 2019-07-16

**Authors:** Ruth L. Dentice, Mark R. Elkins, Jordan Verschuer, Stefan Eberl, Genevieve Dwyer, Peter T. P. Bye

**Affiliations:** 1 Physiotherapy Department, Royal Prince Alfred Hospital, Sydney, Australia; 2 Centre for Education & Workforce Development, Sydney Local Health District, Sydney, Australia; 3 Sydney Medical School, University of Sydney, Sydney, Australia; 4 Department of Molecular Imaging, Royal Prince Alfred Hospital, Sydney, Australia; 5 Physiotherapy Program, Western Sydney University, Sydney, Australia; 6 Department of Respiratory Medicine, Royal Prince Alfred Hospital, Sydney, Australia

**Keywords:** Cystic fibrosis, Body position, Lung deposition, Physical therapy

## Abstract

**Background:**

In people with and without Cystic Fibrosis (CF), does side lying during nebulisation change: the proportion of the dose loaded in the nebuliser that is deposited in the lungs; the uniformity of deposition throughout the lungs; or the apical drug density as a percentage of the drug density in the remaining lung? Do these effects differ depending on the degree of lung disease present?

**Methods:**

A randomised crossover trial with concealed allocation, intention-to-treat analysis and blinded assessors, involving 39 adults: 13 healthy, 13 with mild CF lung disease (FEV_1_ > 80%pred), and 13 with more advanced CF lung disease (FEV_1_ < 80%pred). In random order, 4 mL of nebulised radioaerosol was inhaled in upright sitting and in alternate right and left side lying at 2-min intervals, for 20 min.

**Results:**

Compared to sitting upright, lung deposition and the uniformity of deposition were not significantly altered by side lying in any of the three groups. In sitting, the density of the deposition was significantly less in the apical regions than in the rest of the lung in all participants. Side lying significantly improved apical deposition in healthy adults (MD, 13%; 95% CI, 7 to 19), and in minimal CF lung disease (MD, 4%; 95% CI, 1 to 7) but not in advanced disease (MD, 4%; 95% CI, − 2 to 9).

**Conclusion:**

Alternating between right and left side lying during nebulisation significantly improves apical deposition in healthy adults and in adults with mild CF lung disease, without substantial detriment to overall deposition.

**Trial registration:**

ACTRN12611000674932 (Healthy), ACTRN12611000672954 (CF)

Retrospectively registered 4/7/2011.

**Electronic supplementary material:**

The online version of this article (10.1186/s12890-019-0886-7) contains supplementary material, which is available to authorized users.

## Background

The distribution of deposition of inhaled drugs is influenced by airflow. In sitting, tidal ventilation is preferentially to basal regions [1]. Nebulisation in sitting may under-dose the apical regions [2]. It has been hypothesised that inhalation in alternate side lying will improve homogeneity of drug deposition by dosing each lung (including its apex) when it is dependent [3]. Additionally, many people with Cystic Fibrosis (CF) prefer to nebulise in side lying due to comfort and convenience [4]. A recent study confirmed that the use of an alternate side-lying positioning strategy during inhalation therapy does not prolong nebulisation time [5].

There are however potential disadvantages of a side-lying strategy. Gravitational and biomechanical changes reduce lung volumes and compliance in side lying [6, 7]. Closing volume of the small airways equals or exceeds functional residual capacity as early as 44 years of age, in recumbent positions [8, 9]. Side lying can also decrease alertness [10], negatively impacting inspiratory flows and nebuliser technique. Finally, oropharyngeal pooling may increase the proportion of the loaded drug dose being swallowed in side lying compared to upright sitting. Thus any potential benefits of the side-lying strategy may be outweighed if less drug is deposited in the lungs. Investigation of the strategy’s effect on the pattern of drug deposition is therefore needed to guide clinical practice.

Progression of CF lung disease reduces both lung elasticity and airflow [11]. Therefore, it is possible that the effect of the side-lying strategy would be different in people with mild versus advanced lung disease. Accordingly the research questions were:

1. In people with and without CF, does side lying during nebulisation change the proportion of the dose loaded in the nebuliser that is deposited in the lungs; the uniformity of deposition throughout the lungs; or the apical drug density as a percentage of the drug density in the remaining lung?
2. Do these effects differ depending on the degree of lung disease present?

## Methods

A randomised crossover trial was undertaken to compare two positioning strategies during nebulised delivery of a radioaerosol on 2 days within 1 week, separated by at least one washout day. Adults with CF were recruited from the CF Clinic at Royal Prince Alfred Hospital, Sydney, Australia. Potential participants were excluded if they: had received a lung transplant, were colonised with *Burkholderia cepacia* complex, were not clinically stable; were pregnant; or had hepatomegaly, hepatosplenomegaly, current intestinal obstruction, or significant malignant, neurological or musculoskeletal comorbidities. Adults with CF were recruited in two strata: mild and advanced CF. Mild CF was defined as stable normal lung function with an FEV_1_ and FVC greater than 80% predicted and a normal FEV_1_:FVC ratio, plus minimal upper lobe damage evident on chest radiograph or, if available, on computerised tomographic (CT) scan of the lungs. Advanced CF was defined as stable abnormal (moderate to severe) lung function defined as FEV_1_ less than 80% predicted, with evidence of upper lobe damage on chest radiograph or, if available, on CT scan of the lungs. A third group of healthy adults with an FEV_1_ greater than 80% predicted were recruited from the staff of the hospital.

Testing appointments were scheduled at least 4 h after standard CF morning medications, and not within 1 h of a meal. On both days, participants performed spirometry^1^ in standing according to American Thoracic Society criteria [12]. Healthy participants and participants with mild CF lung disease were required to demonstrate an FEV_1_ greater than 80% predicted that day. Participants with CF were required to have maintained their FEV_1_ within 10% of their best value recorded as an outpatient within the preceding 6 months that day. Participants were then randomised, by flipping a coin, to one of two positioning regimens for their first day: upright sitting, which was maintained throughout the nebulisation period of 20 min; or alternate side lying, with alternation between left and right sides every 2 minutes during the nebulisation period of 20 min.

In an earlier study by our group [5], the two-minute turning regimen and the 20-min total inhalation period were shown to be appropriate to completely nebulise the loaded dose and to ensure equal total nebuliser output while in each side lying position. During the study inhalation periods, all participants were requested to adopt their allocated position and inhale slowly, with a slightly greater volume than at rest, maintaining a lip seal throughout. Inhalation breathing pattern was minimally coached to reflect the spontaneous nebulisation that occurs during unsupervised self-management. When participants returned for their second study day, they adopted the other positioning regimen. Participants with CF were requested to keep their medication regimen, chest physiotherapy and exercise constant during their entire study participation period.

### Outcome measures

#### Radioaerosol inhalation

The standard study inhalation was 4 mL of normal saline mixed with 500 MBq of technetium-99 m diethylene triamine penta-acetic acid (^99m^Tc-DTPA), delivered by an LC Star jet nebuliser (Pari, Germany)^2^ with mouthpiece and nose clip. Delivery of the aerosol was 20 min in duration on each occasion, which was sufficient to deliver a total activity of about 100 MBq. The nebuliser was shielded with a flexible, lead, wrap-around shield. A Y-piece was fitted to the mouthpiece and a filter applied to the expiratory arm to trap exhaled radiation. The nebuliser was driven with 6 L/min of air via the hospital wall supply and a calibrated flow meter.

**Fig. 1:**
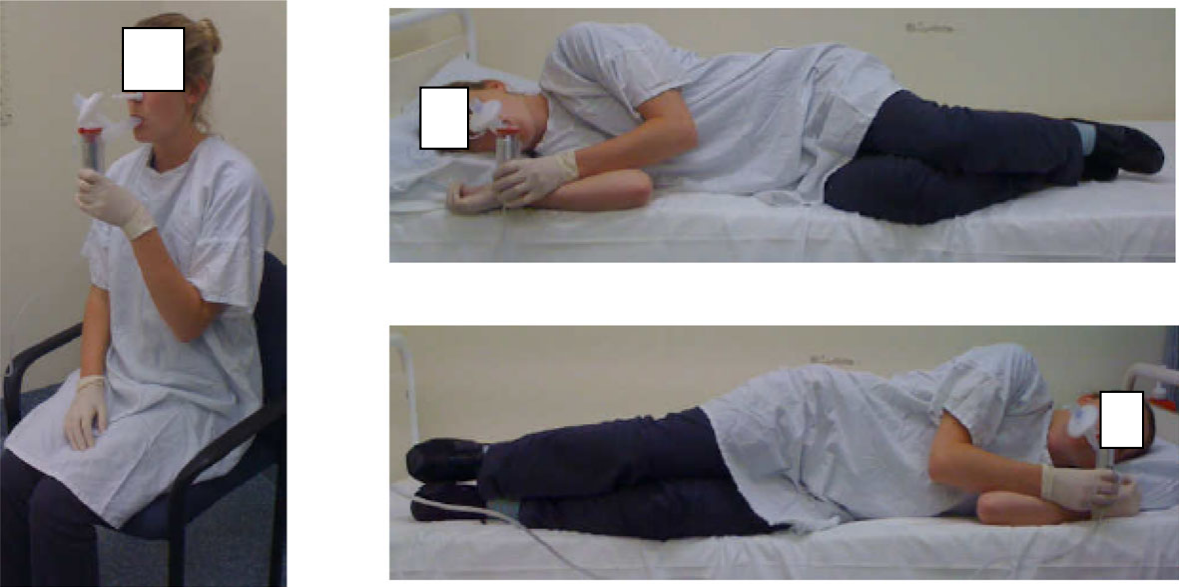
Positioning methods adopted during inhalation with the LC Star jet nebuliser (LC plus nebuliser, Pari, Hamburg, Germany) with mouthpiece, filter and nose clip. Alternate side lying involved dropping the legs over the side of the bed to turn through upright and reposition on the other side every 2 minutes, when instructed by the supervising investigator. (Image with permission)

The nebuliser with tubing, Y-piece and filter were weighed on an electronic balance^3^ before and after the 4 mL dose was loaded; and after the delivery period to allow estimation of the dose delivered to the participant and the residual volume in the nebuliser. The positioning methods adopted during inhalation are pictured in Fig. 1.

#### Imaging of deposition and blinded analysis

**Fig. 2:**
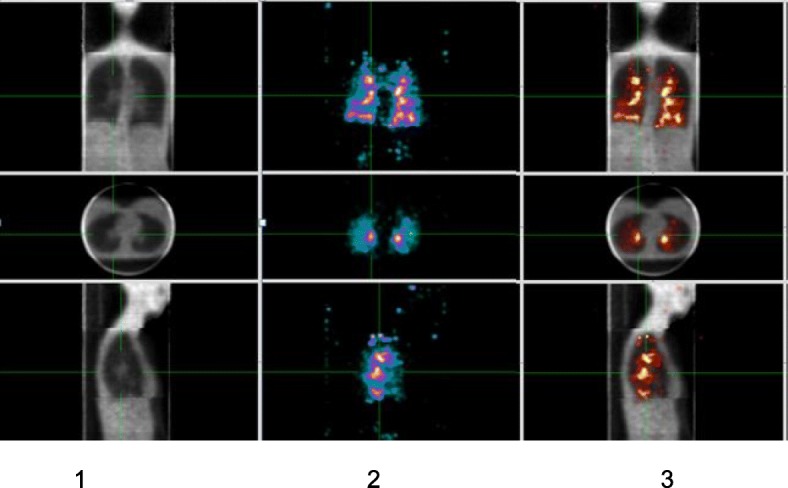
Representative images from one participant during simultaneous emission-transmission dynamic SPECT imaging of radioaerosol deposition: 1. The transmission data is primarily used for attenuation correction of the emission data 2. The emission data records the deposition of the inhaled radioaerosol 3. The transmission data is also used to segment the three-dimensional margin of the lung fields in order to determine the regional distribution of the deposited radioaerosol within the lungs

At the completion of the nebulisation, the participant was positioned supine and head first under a triple-head gamma camera^d^^4^. The camera performed a 20-min deposition scan, incorporating simultaneous emission and transmission acquisitions using a dynamic SPECT imaging protocol well established by our research group [13–16]. Data from the transmission scan were used for attenuation correction of the emission data and also served to segment the three-dimensional margin of the lung fields in order to objectively assess the regional deposition of radioaerosol within the lungs (Fig. 2).

The scan also distinguished regional body deposition: oropharynx versus lungs versus stomach. These data were re-coded before being passed to the investigators to ensure blinding of the body position adopted during each study. For a total delivered activity of 100 MBq, for which approximately 50 to 70% deposits in the lungs, the effective dose to the subject equates to 1.0 to 1.5 mSv per inhalation, giving a total effective dose per participant of 2 to 3 mSv for the complete study. This is safely below the annual radiation dose constraint of 5 mSv per individual in any year for adult volunteers in biomedical research, as mandated by the NSW Radiation Control Regulation [17]. Research procedures were approved by the Ethics Committees of the Sydney Local Health District (RPAH Zone) and the Radiation Safety Officer before commencement. All participants provided written informed consent prior to participating in this study.

### Data analysis

The 3-dimensional map of the lung fields was divided into unit volumes (voxels). The amount of radioactivity in each voxel was calculated. The deposition fraction was calculated as the amount of radioactivity in the lung fields divided by the amount of radioactivity in the nebuliser before inhalation, with correction for radioactive decay. The standard deviation of the radioactivity across all voxels within the margins of the lung fields was used as an index of variability of the pattern of deposition for each participant (variability index). Lower values represent better uniformity of deposition. The ratio of apical:non-apical deposition of radioactivity was determined by the mean number of counts per voxel in the apical one third of the lung field divided by the mean number of counts per voxel in the basal two thirds of the lung field.

The prospectively registered primary outcome was the variability index described above. Our data from initial deposition scans in repeat mucociliary clearance studies in healthy participants indicates that the standard deviation of the uniformity index is 0.18 [18]. In the absence of an existing threshold, we nominated that a 15% improvement in the uniformity index would be the minimum difference that would make the side-lying strategy worthwhile. Adopting a significance level of 0.05 and power of 80%, it was determined, using a commercial sample size calculator^5^, that we would require a sample size of 13 in each of the three groups.

Paired t-tests were used to compare the effect of the two positioning regimens on the deposition fraction, variability index, and ratio of apical: non-apical deposition of radioactivity. Where the data were not normally distributed, a Mann-Whitney test replaced the paired t-test. Correlations between percent predicted FEV_1_ and deposition patterns were investigated with linear regression. Pair-wise independent t-tests were used to compare subgroups with respect to the effect of the positioning on the variability of deposition. All t-tests were reported as a mean difference with a 95% confidence interval.

## Results

### Flow of participants through the study

**Fig. 3:**
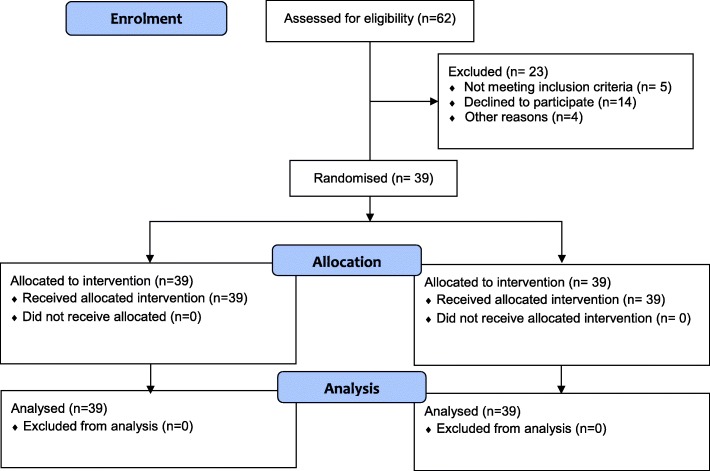
Consort flow diagram

Thirty-nine participants were recruited and completed the study, 13 in each group as outlined in the flowchart in Fig. 3 and Table 1. For individual participant data, see Additional file 1.

**Table 1:** Study participants’ characteristics

Characteristic	Healthy (*n* = 13)	Mild CF (*n* = 13)	Advanced CF (*n* = 13)
Age (y) mean ± SD (range)	28 ± 5 (23 to 40)	27 ± 7 (19 to 41)	30 ± 6 (22 to 41)
FEV_1_ (% pred.) mean ± SD (range)	104 ± 9 (92 to 119)	92 ± 10 (80 to 115)	48 ± 17 (25 to 78)
Gender (M:F)	6:7	6:7	7:6

### Does side lying change the proportion of the dose loaded in the nebuliser that is deposited in the lungs?

In sitting, there was significantly greater lung deposition in mild CF compared to healthy participants mean difference (MD, 5%; 95% CI, 1 to 9). There was also significantly greater lung deposition in mild compared to advanced CF participants (MD, 5%; 95% CI, 1 to 9). The proportion of the dose loaded in the nebuliser that was deposited in the lungs was unaltered by side lying in any group Table 2. The distribution of drug deposition to lung, orophranyx and stomach was also unaltered by side-lying strategy (analyses not shown but raw data is available in the Additional file 1).

**Table 2:** Percentage of the loaded nebuliser dose that deposits in the lungs (mean ± SD), for the three participant groups in sitting and side lying

	Healthy (%)	Mild CF (%)	Advanced CF (%)
Sitting	11 ± 6	16 ± 5	11 ± 5
Side lying	11 ± 6	17 ± 5	11 ± 3

### Does side lying change the uniformity of deposition throughout the lungs?

**Fig. 4:**
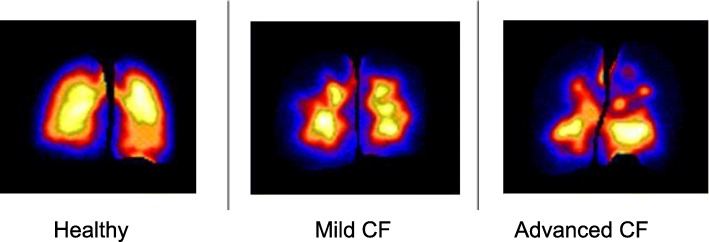
Representative deposition scans for the participant group in sitting

The uniformity index increased with increasing lung disease. The variability of the deposition was not significantly improved or worsened by the side-lying strategy in any of the three groups. The mean variability in lung deposition for each group in the two positioning regimens is presented in Table 3 below with representative scans obtained after the aerosol delivery in sitting in Fig. 4.

**Table 3:** The mean (±SD) variability in lung deposition measured as the standard deviation in counts of radioactivity per unit lung volume in sitting and side lying for the three participant groups, with representative scans of the group participants in sitting

SD counts per voxel	Healthy	Mild CF	Advanced CF
Sitting	1294 ± 920	1920 ± 851	2229 ± 774
Side lying	1250 ± 981	1980 ± 370	1892 ± 486

### Does side lying change the apical drug density?

If drug density was entirely uniform then the apical density per unit volume would be exactly the same as the drug density in the remaining lung per unit volume (100%). In sitting, the density of the deposition was significantly reduced in the apical regions in all groups, to approximately 40% of the density in the non-apical regions. The side-lying strategy significantly improved apical deposition in the healthy adults to 49 ± 14% (MD, 13%; 95% CI, 7 to 20), and in the adults with minimal CF lung disease to 47 ± 7% (MD, 4%; 95% CI, 1 to 7). The mean effect of the side-lying strategy in adults with advanced disease was similar, increasing from 41 ± 20% to 45 ± 21% with side lying, but the variability was greater so this was not statistically significant (95% CI, − 2 to 9) Table 4.

**Table 4:** The apical drug density per unit lung volume as a percentage of the drug density per unit lung volume in the remaining lung; for the three participant groups in sitting and side lying with the mean difference (%) and 95% confidence interval between the two positioning strategies

%	Healthy	Mild CF	Advanced CF
Sitting	36 ± 11	43 ± 8	41 ± 20
Side lying	49 ± 14	47 ± 7	45 ± 21
Mean Difference 95% CI	13 (7 to 19)	4 (1 to 7)	4 (−2 to 9)

### Correlations between change in apical deposition and % predicted FEV_1_

**Fig. 5:**
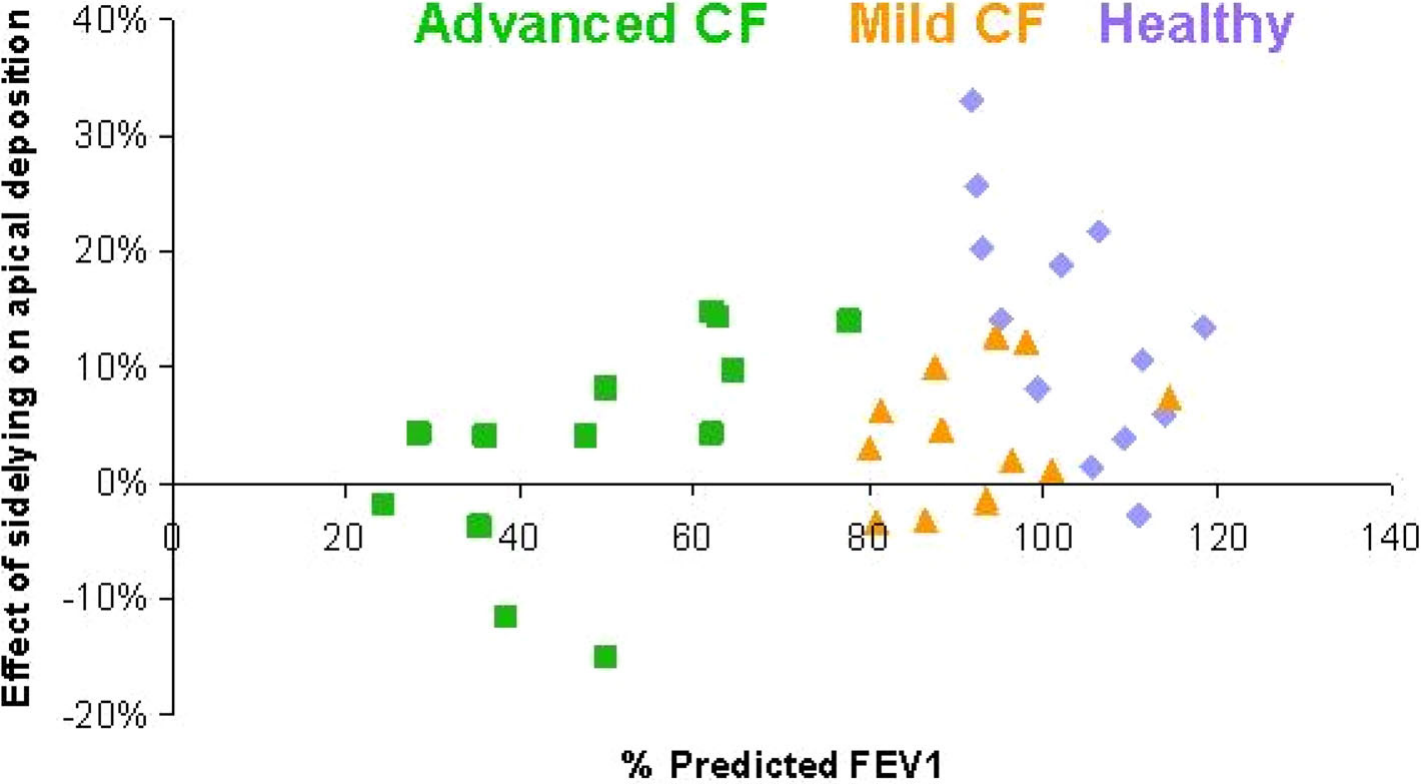
The change in apical deposition in response to the repositioning from upright to side lying for nebulisation. The 13 participants with advanced CF are depicted in green, mild CF in orange and healthy in purple

The change in apical deposition in response to the repositioning from upright to side lying for nebulisation in relation to percent predicted FEV_1_ is represented in Fig. 5. There was no correlation between the change in apical deposition in response to the repositioning from upright to side lying for nebulisation and percent predicted FEV_1_ (*R*^2^ = 0.067).

## Discussion

The percentage of the loaded nebuliser dose that deposited in the lungs was low for all participants, 11 to 16% are consistent with previous and current studies of jet nebulisers [2, 19, 20]. Healthy participants had significantly lower lung deposition than participants with mild CF in both positioning regimens. Participants with CF were very familiar with nebulised therapies in comparison to healthy naive participants who reported that 20 min of inhalation on a nebuliser was more challenging than they expected. The distribution of drug deposition to lung, orophranyx and stomach was unaltered by side-lying strategy.

In sitting, adults with advanced CF had almost twice as much variability in lung drug deposition compared to healthy adults of similar age. With increasing lung disease there is increasing variability in lung deposition of a nebulised drug. This finding is consistent with previous work involving deposition scanning in this patient population [3, 4, 21–23]. The variability of deposition was not substantially altered by side lying in either group. Participants with advanced CF demonstrated the greatest response with a MD in the variability index of 337 (95% CI, − 186 to 860). This equates to a 7% change, which is half that of our proposed minimum clinically worthwhile difference of 15%; nevertheless, the positive trend provides further reassurance that taking a nebuliser in side lying does not substantially compromise drug deposition.

Statistically the side-lying positioning strategy can improve apical deposition. Our best estimate of the effect is a positive one in all three participant groups (Healthy: 13% better (95%CI: 7 to 19) Mild CF: 4% better (95%CI: 1 to 7) Advanced CF: 4% better (95%CI: − 2 to 9). It is difficult to estimate the clinical impact of this small shift of 4 % towards greater apical deposition in CF participants over the course of multiple nebulised drug doses. Permitting people with CF to have the flexibility to nebulise in side lying may improve treatment adherence in patients who prefer this regimen. The effect of regular use of this positioning regimen could be further examined in a longitudinal trial.

The side-lying strategy was only effective at reversing the deficiency in apical deposition in the subgroups of participants who had well preserved lung function. This suggests that the side-lying strategy may be more helpful in children with CF, or in respiratory conditions where lung function is well preserved, such as pneumocystis pneumonia [24].Additionally, the delivery rate of inhaled medications should be further investigated in mesh and adaptive delivery nebulisers and with drugs of greater viscosity.

## Conclusion

There is no negative impact on lung deposition if adults with CF nebulise in alternate side lying. Adults with CF have almost twice as much variability in the density of nebulised drug deposition across the lung fields compared to healthy adults, which is unaltered with inhalation in side lying. Positioning in side lying can improve apical deposition in healthy lungs and to a lesser extent in mild CF lung disease. Given many patients prefer to nebulise in side lying due to comfort and convenience, permitting nebulisation in side lying may improve treatment adherence without adversely affecting deposition, provided side lying on each side is given equal time.

## Additional file

Additional file 1: Individual participant data (XLSX 18 kb)

## Data Availability

The datasets used and/or analysed during the current study are available from the corresponding author on reasonable request.

## Abbreviations

^99m^Tc-DTPA: Technetium-99 m diethylene triamine penta-acetic acid
CF: Cystic Fibrosis
FEV_1_: Forced expiration in the first second
FVC: Forced vital capacity
MD: Mean difference

## Footnotes

1 Easy-One, NDD Medical Technologies, Andover, USA

2 LC plus nebuliser, Pari, Hamburg, Germany

3 1206MP Scale, Sartorius, Cambridge, UK

4 Trionix XLT Triple Head Gamma Camera, Trionix, Twinsburg, USA

5 PS Power & Sample Size Calculator, Visual Components, Lake Orion, USA.
